# The clinical relevance of humoral immune responses to Globo H-KLH vaccine adagloxad simolenin (OBI-822)/OBI-821 and expression of Globo H in metastatic breast cancer

**DOI:** 10.1136/jitc-2021-004312

**Published:** 2022-06-22

**Authors:** Jung-Tung Hung, I-Ju Chen, Shir-Hwa Ueng, Chiun-Sheng Huang, Shin-Cheh Chen, Mu-Yi Chen, Yung-Chang Lin, Chun-Yen Lin, Michael J Campbell, Hope S Rugo, Alice L Yu

**Affiliations:** 1 Institute of Stem Cell and Translational Cancer Research, Chang Gung Memorial Hospital at Linkou, Taoyuan, Taiwan; 2 OBI Pharma Inc, Taipei, Taiwan; 3 Department of Pathology, Chang Gung Memorial Hospital at Linkou, Taoyuan, Taiwan; 4 Department of Surgery, National Taiwan University Hospital and National Taiwan University College of Medicine, Taipei, Taiwan; 5 Department of General Surgery, Chang Gung Memorial Hospital at Linkou, Taoyuan, Taiwan; 6 Department of Internal Medicine, Chang Gung Memorial Hospital at Linkou, Taoyuan, Taiwan; 7 Department of Gastroenterology and Hepatology, Chang Gung Memorial Hospital at Linkou, Taoyuan, Taiwan; 8 Department of Surgery, Division of Surgical Oncology, University of California San Francisco, San Francisco, California, USA; 9 Helen Diller Family Comprehensive Cancer Center, University of California San Francisco, San Francisco, California, USA; 10 Department of Pediatrics, University of California San Diego School of Medicine, La Jolla, California, USA; 11 Graduate Institute of Biomedical Sciences, Chang Gung University, Taoyuan, Taiwan

**Keywords:** Antigens, Tumor-Associated, Carbohydrate, Breast Neoplasms, Clinical Trials as Topic, Immunity, Humoral, Immunization

## Abstract

An international randomized phase II trial of Globo H (GH) vaccine, adagloxad simolenin/OBI-821 in 349 patients with metastatic breast cancer showed longer progression-free survival (PFS) in vaccinated patients who developed anti-Globo H (anti-GH) IgG than those who did not and the placebo group. The impacts of anti-GH IgM and GH expression on peak anti-GH IgG and clinical outcome were further evaluated. The titers of anti-GH IgG and IgM were determined by ELISA. GH expression in tumor was examined by immunohistochemical staining. Immunophenotyping was conducted by flow cytometry. Adagloxad simolenin elicited anti-GH IgM which peaked at titers ≥1:80 between weeks 5 and 13. The mean anti-GH IgG titer peaked at week 41 and decreased thereafter on the completion of vaccination. One log increase in peak IgM was associated with 10.6% decrease in the HR of disease progression (HR: 0.894, 95% CI: 0.833 to 0.960, p=0.0019). Patients with anti-GH IgM ≥1:320 within first 4 weeks after vaccination had significantly higher maximum anti-GH IgM (p<0.0001) and IgG titers (p<0.0001) than those with <1:320. Moreover, the median PFS appears to be longer for patients with anti-GH IgM ≥1:320 within first 4 weeks than those with anti-GH IgM titer <1:320 (11.1 vs 7.3 months, p=0.164), but not statistically significant. Among patients with H score ≥80 for GH expression by immunohistochemistry, the vaccination group (n=42) seemed to have better PFS than the placebo group (n=23) (HR=0.59; 95% CI: 0.32 to 1.10, p=0.10), but the difference did not reach statistical significance. In addition, peak levels of anti-GH IgM were higher in patients who had lower percentage of activated regulatory T cells (Treg cells; CD4^+^CD45RA^-^Foxp3^high^) at baseline than those who had higher activated Treg cells (p=0.042). This study demonstrates that adagloxad simolenin induced both IgG and IgM antibodies against GH. Anti-GH IgM ≥1:320 within first 4 weeks or low activated Treg cells at baseline may help to select patients who are likely to produce a higher level of GH-specific IgM and IgG in the future.

## Background

Globo H (GH) is a glycosphingolipid with a hexasaccharide moiety (Fucα1→2Galβ1→3GalNAcβ1→3Galα1→4Galβ1→4Glcβ1). GH is highly expressed on a variety of cancers including breast, colon, ovarian, gastric, pancreatic, lung, and prostate cancers.^1^ Its absence in vital organs and limited expression at immune-privileged luminal surfaces of normal glandular tissues makes GH an ideal target of cancer immunotherapy.^1^ Moreover, the finding of GH on cancer stem cells lends further support for targeting it for tumor eradication.^2^

Adagloxad simolenin (AS) is a glycoconjugate composed of GH covalently linked to a carrier protein, keyhole limpet hemocyanin (KLH). OBI-821 derived from the bark of the *Quillaja saponaria* Molina tree is a saponin-based adjuvant. Phase I clinical trials of active immunotherapy with AS in prostate and late-stage breast cancers demonstrated its safety and disease stabilization in some patients.^3 4^ We conducted an international randomized phase II trial of AS with OBI-821 in 349 patients with metastatic breast cancer (MBC). Although there was no significant difference in the progression-free survival (PFS) between the vaccinated (95% CI: 6.5 to 10.9) and the placebo group (95% CI: 7.3 to 11.3), PFS was significantly longer in the vaccinated patients who developed anti-Globo H (anti-GH) IgG titer ≥160 (PFS 95% CI: 9.3 to 17.6) than those who did not (95% CI: 3.7 to 5.6, p<0.0001) and the placebo group (95% CI: 7.3 to 11.3, p=0.002).^5^ AS/OBI-821 treatment was well tolerated. The most common treatment-related adverse events were grades 1–2 injection site reactions (56.7%) and fever (20.1%). Herein, we report the impacts of anti-GH IgM immune responses to AS/OBI-821 and tumor expression of GH on peak IgG titers and clinical outcome.

## Materials and methods

### Patients

This randomized double-blind study, OPT-822-001, was conducted across 40 centers in five countries as described previously.^5^ Briefly, patients with MBC of any molecular subtype and two or less prior progressive disease events with stable/responding disease after the last anticancer regimen were randomized (2:1) to receive AS/OBI-821 or placebo, subcutaneously for nine doses with low-dose cyclophosphamide (CY). A total of 349 patients were randomized and 348 patients received study drug. The study was approved by the independent ethics committee or institutional review board at each site. Written informed consents were obtained from all patients enrolled.

### Study treatment

AS/OBI-821 (30 µg/100 µg) or placebo was administered by subcutaneous injection on weeks 1, 2, 3, 5, 9, 13, 17, 25, and 37 for a total of nine doses or until disease progression. All patients received CY 300 mg/m^2^ intravenously 3 days prior to each dose of AS/OBI-821 on weeks 1, 5, 9, 13, 17, 25, and 37. The treatment schema is shown in online supplemental figure S1.

10.1136/jitc-2021-004312.supp1 Supplementary data

**Figure 1:**
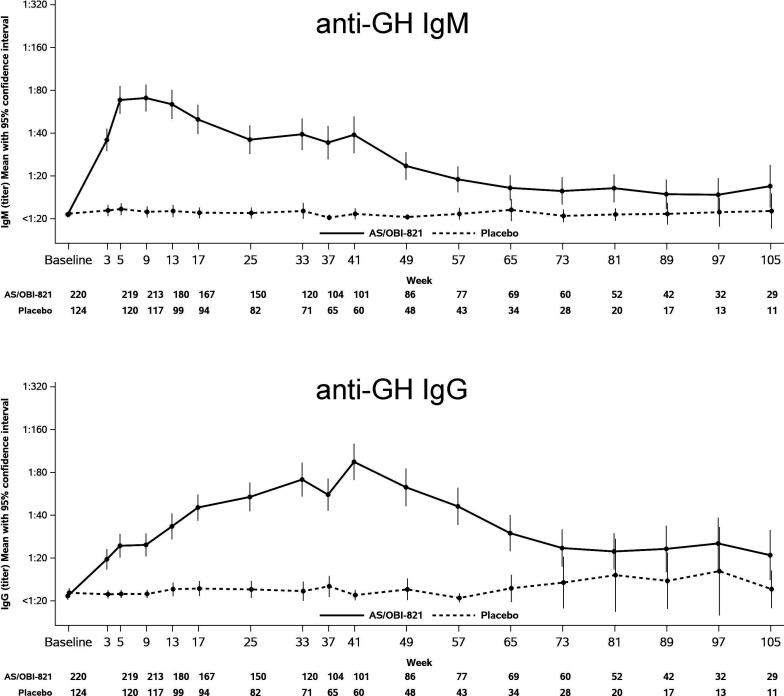
IgM and IgG antibody titers against Globo H (GH)-ceramide after immunization with adagloxad simolenin/OBI-821. Serum from patients was collected before (baseline) and after immunization. Anti-GH antibodies were determined by ELISA. Mean with 95% confluence interval was presented. The numbers below the figure indicate the number of subjects evaluated at the specific time point.

### ELISA for the determination of anti-Globo H titers

Serum samples were collected for the measurement of anti-GH titers by ELISA on weeks 1, 3, 5, 9, 13, 17, 25, 33, 37, 41, and every 8 weeks thereafter for up to 2 years or until disease progression. ELISA assay for measuring the tiers of anti-GH was performed as described previously^3^ and provided in online supplemental figure S1. The optical density (OD) values obtained from the GH-coated plates were subtracted with the OD values from uncoated plates. The cut-off value was calculated by adding 0.1 to the mean OD value of negative control (secondary antibody only).^3^ The highest serum dilution with an absorbance more than or equal to cut-off value was recorded as the antibody titer. If the absorbance of the sample in 20-fold dilution is less than the cut-off value, the titer of the sample was recorded as 0.

### Immunohistochemistry

Tumor samples were collected at the time of diagnostic surgery. Sections (3 µm) of archival paraffin blocks were used to determine the GH expression as described previously.^6^ The percentage and intensity of GH-positive cells in tumor tissue were read by pathologists. H score was calculated by the following equation: percentage of weak intensity×1+percentage of moderate intensity×2+percentage of strong intensity×3.

### Immunophenotyping

Heparinized blood was collected from 71 patients in University of California in San Francisco (UCSF) and 28 patients in Linkou Chang Gung Memorial Hospital (CGMH-LK) at baseline (prevaccine, day 1), day 4 (3 days post-CY and prior to first vaccination), week 5 and week 13 before CY, and week 41 (online supplemental figure S1). Peripheral blood mononuclear cells (PBMCs) were stained with antibodies to CD3 (SK7, BD Biosciences), CD4 (SK3, BD Biosciences), CD45RA (HI100, BD Pharmingen), CD25 (M-A251, BD Pharmingen), CD56 (B159, BD Biosciences), CD107a (H4A4, BD Biosciences) or CD69 (FN50, BD Biosciences) for 30 min at 4°C. After fixation and permeabilization, cells were stained with anti-FOXP3 (PCH101, eBioscience) for 30 min. For the detection of intracellular cytokine production, aliquots of PBMC were incubated with 10% fetal bovine serum (FBS)/RPMI (Roswell Park Memorial Institute) 1640 medium overnight at 37°C and then stimulated with 50 ng/mL PMA plus 1 µg/mL ionomycin in the presence of Golgi-Stop (BD Pharmingen) for 4 hours and then stained with antibodies against interleukin (IL)-17a (N49-653), IL-4 (MP4-25D2), interferon-γ (B27), or CD8 (RPA-T8, BD Pharmingen). The corresponding negative controls were mouse IgG1-PerCP, IgG2b-FITC (fluorescein isothiocyanate), IgG1-PE, IgG1-PECy5 (phycoerythrin cyanine 5), IgG1-APC (allophycocyanin), IgG1-FITC, and rat IgG2a-APC.

### Statistical analysis

For the analysis of GH antibody titers, the geometric means and the associated two-sided CIs were derived by calculating means and CIs on the natural log scale based on the t-distribution, and then extrapolating the results. Titers below the LLOQ (lower limit of quantitation) or denoted as 0 were set to 0.5×20 for analysis. PFS was evaluated using the Kaplan-Meier method and group comparison (HR and p value) estimated from the stratified Cox proportional hazards model. For immunophenotyping analysis, the biomarker cut-off value was based on its relationship to PFS, and the time-dependent receiver operating characteristic curve analysis was used to determine the prognostic accuracies of biomarker. P-value was the group comparison on maximum IgM level based on t-test. For all test results, two-sided p values were reported and interpreted as significant at 5%. P values were not corrected for multiple testing because of the exploratory nature of this study. Data analysis and visualization were conducted based on statistical tool SAS V.9.4.

## Results

### AS/OBI-821 induced both IgM and IgG antibodies against Globo H in patients with MBC

**Figure 2:**
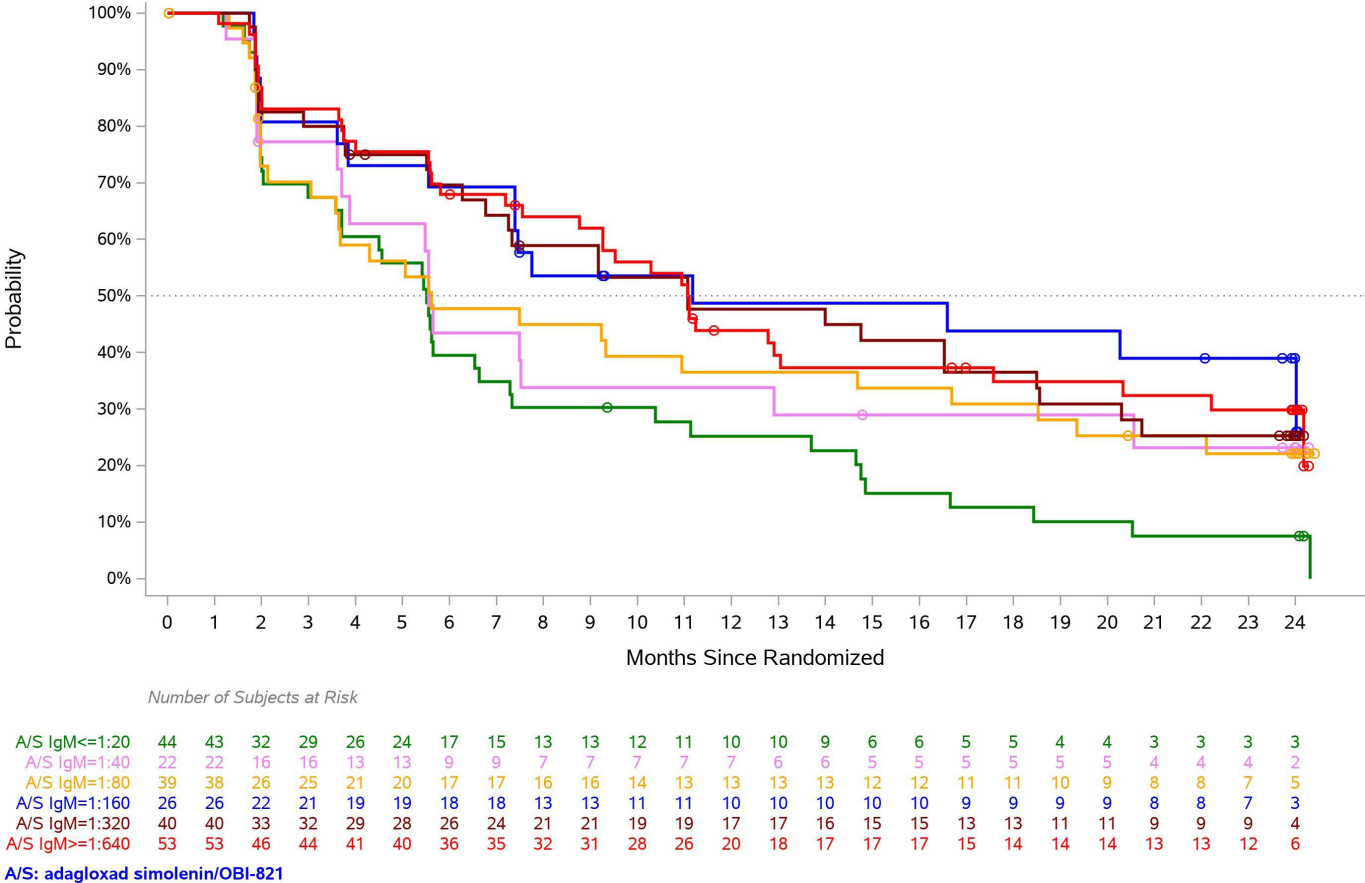
Globo H IgM response induced by adagloxad simolenin correlate to progression-free survival (PFS). PFS for adagloxad simolenin/OBI-821 recipients were presented according to anti-Globo H IgM titer level. Each curve represents a group of patients with their maximum anti-Globo H IgM antibody titers at any time during the study reaching the specified level. Green: 1:20, pink: 1:40, yellow: 1:80, blue: 1:160, purple: 1:320, red: 1:640.

AS/OBI-821 elicited anti-GH IgM responses (titer ≥1:20) in 199 out of 224 patients which reached a peak titer of ≥1:80 between weeks 5 and 13 (after three to five injections) and then declined. The anti-GH IgG titer ≥1:20 was detected in 182/224 of patients which peaked at week 41 (after nine injections) and decreased once vaccination was completed (figure 1). Previously, we reported higher anti-GH IgG levels correlated with better PFS.^5^ Similar correlation was observed in anti-GH IgM responses (figure 2). Based on Cox model under linear trend in IgM on PFS, a one log increase in IgM was associated with a 10.6% decrease in the hazard of disease progression (HR of 0.894 with a 95% CI of 0.833 to 0.960, p=0.0019). Thus, greater peak anti-GH IgM and IgG levels might contribute to longer PFS. More importantly, those with anti-GH IgM ≥1:320 within first 4 weeks after vaccination not only exhibited substantially higher peak GH IgM than those with <1:320 (mean peak titers 746.6 vs 68.8; p<0.0001) but also mounted significantly higher maximum anti-GH IgG (222.6 vs 85.0; p<0.0001) in response to AS/OBI-821 (table 1). These findings implied that anti-GH IgM within first 4 weeks after vaccination could serve as a predictor of subsequent GH IgM and IgG responses, both of which showed positive correlation with clinical outcome. In addition, the median PFS for patients who mounted anti-GH IgM responses ≥1:320 within first 4 weeks after vaccination seemed to be longer (n=161, 11.1 months, 95% CI: 7.56 to 14) than those who did not (n=63, 7.3 months, 95% CI: 5.59 to 9.33, p=0.1635; table 1), although it did not reach statistical significance.

**Table 1:** Median progression-free survival (PFS) and peak mean anti-Globo H (anti-GH) IgM and IgG of the subject groups with anti-GH IgM ≥1:320 and <1:320 within 4 weeks after vaccination

	Adagloxad simolenin/OBI-821 group with IgM level within first 4 weeks		
	<1:320 (n=161)	≥1:320 (n=63)	P value
Median PFS (month)	7.3	11.1	0.1635*
Peak mean IgM	68.8	746.6	<0.0001†
Peak mean IgG	85	222.6	<0.0001

*Log-rank test

†t-test

### Correlation between Globo H expression and PFS to AS in MBC

A post hoc exploratory analysis was used to evaluate the association between GH expression of tumors and PFS in patients treated with AS/OBI-821 or placebo. For patients with immunohistochemistry H score ≥80, the median PFS was 12.8 months (95% CI 5.6 to 19.4) in the AS/OBI-821 group (n=42) as compared with 9.2 months (95% CI 1.9 to 12.5) in the placebo group (n=23, online supplemental figure S2), and the PFS rate at 1.5 years was 44% and 10%, respectively (HR: 0.59, 95% CI 0.32 to 1.10, p=0.10). Thus, there appears to be a potential PFS benefit of AS/OBI-821 vaccine for subjects with higher GH expression. However, the difference did not reach statistical significance, likely due to the small number of patients with H score >80 in this study.

### Globo H expression at metastatic site versus primary site

Thirty-two subjects with paired primary tumor and metastatic lesions were evaluated for their GH expression. Eighteen of 32 (56.3%) subjects showed higher GH expression on primary tumors than their metastatic counterparts, including 8 patients (25%) whose tumors expressed GH only at primary site, but not metastatic lesions. On the other hand, 28.1% (9/32) of the subjects showed greater GH expression on metastatic tumors than their respective primary tumors. The remaining 15.6% (5 out of 32) of the subjects have equivalent expression of GH on primary and metastatic tumors. As shown in online supplemental figure S3, GH expression showed a trend of correlation between primary and metastatic tumors of the same patients, but did not reach statistical significance (Pearson correlation coefficient: 0.306, p=0.0885). These results should be interpreted with caution, given the limited number of metastatic tumors available for the study.

**Figure 3:**
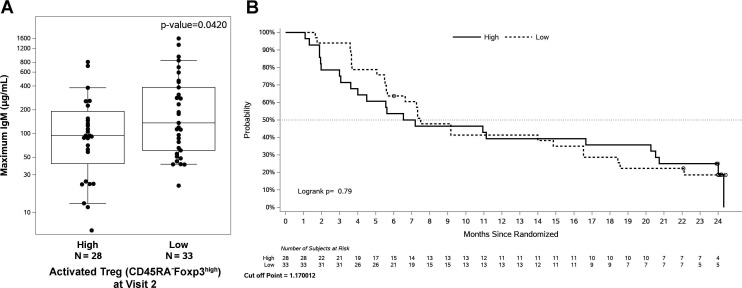
Correlation of activated regulatory T (Treg) cells with maximal anti-Globo H (anti-GH) IgM levels and progression-free survival. Peak anti-GH IgM levels in vaccinated subjects with low and high activated baseline Treg. Peripheral blood mononuclear cells (PBMCs) from vaccinated patients were isolated at baseline for the determination of activated Treg populations (CD4^+^CD45RA^-^Foxp3^+^) by fluorescence-activated cell sorting (FACS) analysis. (A) Box plots are presented as the 10th percentile low and the 90th percentile high (whiskers), center line as the median, and bounds of box mark the first and third quartiles. (B) Progression-free survival of patients with high and low activated Treg cells.

### Low-dose cyclophosphamide enhanced CD107a^+^ natural killer (NK) cell population but had no impact on Treg

All patients received CY 300 mg/m^2^ administered intravenously at weeks 1, 5, 9, 13, 17, 25, and 37, 3 days prior to each dose of AS/OBI-821 or placebo. Blood samples from 71 patients in UCSF and 28 patients in CGMH-LK were collected at baseline (before first CY), day 4 (3 days after first CY), week 5 (before second CY), week 13 (intermediate time point), and week 41 (end of study) to evaluate changes in circulating immune effector cell subpopulations. No significant changes in T helper cells, including Th1 (CD4^+^IFN-γ^+^), Th2 (CD4^+^IL-4^+^), and Th17 (CD4^+^IL-17^+^); cytotoxic T cells (CD3^+^CD8^+^IFN-γ^+^); natural killer T (NKT, CD3^+^CD56^+^) cells, and Treg cells, including total Treg (CD4^+^Foxp3^+^, CD4^+^CD25^+^, or CD4^+^CD25^+^Foxp3^+^), resting Treg (CD45RA^+^Foxp3^low^), activated Treg (CD45RA^-^Foxp3^high^) and non-suppressive Treg (CD45RA^-^Foxp3^low^) were observed over time (online supplemental figure S4). CD107a has been shown to be a marker of activated NK cells. Interestingly, low-dose CY appeared to enhance CD107a^+^ NK cell population (p=0.038, paired t-test) but not the number of total NK cells nor Treg cell populations in PBMC (online supplemental figure S5).

### AS/OBI-821-vaccinated subjects with lower baseline Treg mounted higher anti-GH IgM responses

Activated Treg (CD4^+^CD45RA^-^Foxp3^high^) in colorectal cancer tissues has been reported to be higher in patients with metastasis.^7^ A post hoc analysis was used to evaluate the correlation between circulating immune cells at baseline or 3 days after first CY and the humoral response to AS/OBI-821. The patients who had low percentage of activated Treg cell population at baseline mounted higher peak anti-GH IgM (164.7±3.16 µg/mL) than those who had high activated Treg cells (87.6±3.39 µg/mL, p=0.042, t-test), but it had no impact on PFS (figure 3). The number of activated Treg cells 3 days post-CY did not correlate with either anti-GH responses or PFS. The result implied that intrinsic Treg cells may have adverse impact on the immune response to AS/OBI-821.

## Discussion

In this study, we showed that a carbohydrate antigen vaccine AS/OBI-821 could induce both GH-specific IgM and IgG antibodies in patients with advanced breast cancer,^5^ suggesting its ability to induce isotype switching from IgM to IgG. Recent studies have demonstrated that glycotope linked to peptides can be recognized by T cells. After processing of polysaccharide coupled to a carrier protein in antigen-presenting cells, a carbohydrate epitope linked to peptide is presented by major histocompatibility class II to stimulate carbohydrate-specific CD4^+^ T cells to produce IL-2 and IL-4 cytokines, which are essential for providing T cell help to antibody-producing B cells.^8^ Alternatively, follicular B cells may take up glycol conjugates via their glycotope-specific B-cell receptor, process the carrier protein moieties and present the resulting peptides on major histocompatibility class II molecules, to engage peptide-specific T cells.^9^ The exact mechanism of how AS induces class switch remains to be elucidated.

So far, all Food and Drug Administration-approved therapeutic anticancer monoclonal antibodies belong to IgG class, even though the pentameric IgM is more avid in antigen binding. This is largely due to the bottlenecks of manufacturing recombinant IgM. Recently, recombinant technology has been developed to overcome challenges in IgM production.^10^ For example, monoclonal antibody 216, an IgM targeting a linear B-cell lactosamine antigen, has shown efficient binding to leukemic blasts and favorable early responses in a phase I trial of patients with relapsed or refractory B-cell acute lymphoblastic leukemia.^11^ PAT-SM6, a monoclonal IgM against glucose regulated protein 78, is well-tolerated and shows modest clinical activity in a phase I trial of patients with relapsed or refractory multiple myeloma.^12^ A bispecific IgM anti-CD20, IGM-2323, CD20XCD3, which consists of 10 binding units for CD20 and 1 binding unit for CD3 displays better binding on cancer cells that express relatively lower amounts of CD20 on the cancer cell surface in vitro.^13^ A phase I trial of CD20XCD3 in patients with relapsed/refractory B cell non-Hodgkin’s lymphoma is ongoing (NCT04082936). In addition, IgM pertuzumab can inhibit the proliferation in HER2 (human epidermal growth factor receptor 2) overexpressing cells more effectively than its IgG1 counterpart.^14^ In this study, we found that patients with higher anti-GH IgM levels had better PFS outcome, suggesting that vaccine-induced GH IgM antibodies could play a role in the anticancer activity. Of note, our observation that anti-GH IgM ≥1:320 within first 4 weeks was associated with higher peak IgM and IgG titers and clinical benefits suggests that early IgM response to AS/OBI-821 may serve as a valuable screening test for patient selection in the future clinical trials of AS/OBI-821 vaccine.

The regulatory approval of anti-GD2 for the treatment of neuroblastoma has set the stage for cancer-associated carbohydrate antigens as new class of targets for cancer immunotherapy.^15^ In this context, the ability of AS/OBI-821 vaccine to induce GH-specific IgM and IgG antibodies, which displayed the biological functions of complement-dependent cytotoxicity (CDC) and antibody-dependent cellular cytotoxicity (ADCC),^5^ and the prevalence of GH expression in cancer with limited expression in normal tissues make AS/OBI-821 vaccine a promising new carbohydrate-targeted immunotherapeutics. In addition, these antibodies may exert anticancer effects by neutralizing Globo H ceramide (GHCer) shed from cancer cells into the tumor microenvironment.^6^ It has been reported that shed GHCer can be incorporated by endothelial cells to promote angiogenesis^6^ and by tumor-infiltrating lymphocytes to inhibit the activation of T and B cells.^16^ In this clinical trial, GH-specific IgG antibodies reached peak titer at week 41, 3 weeks after the last (ninth) injection, and then declined. This suggests that AS/OBI-821 is unable to sustain anti-GH IgG response without continuous vaccination. Thus, future clinical trial should consider extension of the vaccination schedule beyond week 41 to maintain anti-GH antibody at high titers so as to improve anticancer efficacy and prolong patient survival.

We found that patients with higher GH expression in breast tumor tissue may have a potential PFS benefit from AS/OBI-821 vaccine. Given the relatively small sample size, these data should be interpreted with caution. If confirmed in a larger cohort, GH expression levels in tumor may serve as a biomarker for selecting patients for AS/OBI-821 vaccine therapy, similar to the use of programmed cell death 1 ligand 1 (PD-L1) expression for anti-programmed cell death protein 1 (PD-1) therapy.^17 18^

In summary, we showed that greater peak anti-GH IgM levels were associated with longer PFS of patients, similar to the significant correlation of higher anti-GH IgG levels with better PFS, as we previously reported.^5^ Moreover, our findings suggest the potential value of anti-GH IgM ≥1:320 within first 4 weeks and higher GH expression on tumor in selecting patients who are likely to benefit from AS/OBI-821 in the future, if confirmed in the ongoing global phase III trial of AS/OBI-821.

## Data Availability

Data sharing not applicable as no datasets generated and/or analysed for this study. All data relevant to the study are included in the article or uploaded as supplemental information.

## References

[1] Zhang S, Zhang HS, Cordon-Cardo C, et al. Selection of tumor antigens as targets for immune attack using immunohistochemistry: II. blood group-related antigens. Int J Cancer 1997;73:50–6. 10.1002/(sici)1097-0215(19970926)73:1&lt;50::aid-ijc9&gt;3.0.co;2-0 9334809

[2] Chang W-W, Lee CH, Lee P, et al. Expression of globo H and SSEA3 in breast cancer stem cells and the involvement of fucosyl transferases 1 and 2 in globo H synthesis. Proc Natl Acad Sci U S A 2008;105:11667–72. 10.1073/pnas.0804979105 18685093 PMC2575305

[3] Gilewski T, Ragupathi G, Bhuta S, et al. Immunization of metastatic breast cancer patients with a fully synthetic globo H conjugate: a phase I trial. Proc Natl Acad Sci U S A 2001;98:3270–5. 10.1073/pnas.051626298 11248068 PMC30643

[4] Slovin SF, Ragupathi G, Adluri S, et al. Carbohydrate vaccines in cancer: immunogenicity of a fully synthetic globo H hexasaccharide conjugate in man. Proc Natl Acad Sci U S A 1999;96:5710–5. 10.1073/pnas.96.10.5710 10318949 PMC21925

[5] Huang C-S, Yu AL, Tseng L-M, et al. Globo H-KLH vaccine adagloxad simolenin (OBI-822)/OBI-821 in patients with metastatic breast cancer: phase II randomized, placebo-controlled study. J Immunother Cancer 2020;8:e000342. 10.1136/jitc-2019-000342 32718986 PMC7380846

[6] Cheng J-Y, Wang S-H, Lin J, et al. Globo-H ceramide shed from cancer cells triggers translin-associated factor X-dependent angiogenesis. Cancer Res 2014;74:6856–66. 10.1158/0008-5472.CAN-14-1651 25281721

[7] Lin Y-C, Mahalingam J, Chiang J-M, et al. Activated but not resting regulatory T cells accumulated in tumor microenvironment and correlated with tumor progression in patients with colorectal cancer. Int J Cancer 2013;132:1341–50. 10.1002/ijc.27784 22907255

[8] Avci FY, Li X, Tsuji M, et al. A mechanism for glycoconjugate vaccine activation of the adaptive immune system and its implications for vaccine design. Nat Med 2011;17:1602–9. 10.1038/nm.2535 22101769 PMC3482454

[9] Lucas AH, Apicella MA, Taylor CE. Carbohydrate moieties as vaccine candidates. Clin Infect Dis 2005;41:705–12. 10.1086/432582 16080094 PMC7107877

[10] Chromikova V, Mader A, Steinfellner W, et al. Evaluating the bottlenecks of recombinant IgM production in mammalian cells. Cytotechnology 2015;67:343–56. 10.1007/s10616-014-9693-4 24615530 PMC4329305

[11] Liedtke M, Twist CJ, Medeiros BC, et al. Phase I trial of a novel human monoclonal antibody mAb216 in patients with relapsed or refractory B-cell acute lymphoblastic leukemia. Haematologica 2012;97:30–7. 10.3324/haematol.2011.045997 21993685 PMC3248928

[12] Rasche L, Duell J, Castro IC, et al. GRP78-directed immunotherapy in relapsed or refractory multiple myeloma - results from a phase 1 trial with the monoclonal immunoglobulin M antibody PAT-SM6. Haematologica 2015;100:377–84. 10.3324/haematol.2014.117945 25637055 PMC4349277

[13] Baliga R, Li K, Manlusoc M, et al. High avidity IgM-Based CD20xCD3 bispecific antibody (IGM-2323) for enhanced T-cell dependent killing with minimal cytokine release. Blood 2019;134:1574. -. 10.1182/blood-2019-131650

[14] Samsudin F, Yeo JY, Gan SK-E, et al. Not all therapeutic antibody isotypes are equal: the case of IgM versus IgG in Pertuzumab and Trastuzumab. Chem Sci 2020;11:2843–54. 10.1039/C9SC04722K 32206268 PMC7069520

[15] Yu AL, Gilman AL, Ozkaynak MF, et al. Anti-GD2 antibody with GM-CSF, interleukin-2, and isotretinoin for neuroblastoma. N Engl J Med 2010;363:1324–34. 10.1056/NEJMoa0911123 20879881 PMC3086629

[16] Tsai Y-C et al. A prevalent cancer associated glycan, globo H ceramide, induces immunosuppression by reducing Notch1 signaling. J Cancer Sci Ther 2013;05:264–70. 10.4172/1948-5956.1000215

[17] Topalian SL, Hodi FS, Brahmer JR, et al. Safety, activity, and immune correlates of anti-PD-1 antibody in cancer. N Engl J Med 2012;366:2443–54. 10.1056/NEJMoa1200690 22658127 PMC3544539

[18] Taube JM, Klein A, Brahmer JR, et al. Association of PD-1, PD-1 ligands, and other features of the tumor immune microenvironment with response to anti-PD-1 therapy. Clin Cancer Res 2014;20:5064–74. 10.1158/1078-0432.CCR-13-3271 24714771 PMC4185001

