# Supplementary material for: The clinical relevance of humoral immune responses to Globo H-KLH vaccine adagloxad simolenin (OBI-822)/OBI-821 and expression of Globo H in metastatic breast cancer
Source: J Immunother Cancer. 2022 Jun 22;10(6):e004312. doi: 10.1136/jitc-2021-004312 (PMC9226869; doi:10.1136/jitc-2021-004312)
Supplement: Supplementary data [file jitc-2021-004312supp001.pdf]

Supplemental Materials and Methods

ELISA

Globo H-ceramide (0.2 μg in 95% ethanol/well, 50 μL) was coated in each well of 96-well plates (cat# 442404, NUNC) at room temperature (RT) overnight. Non-specific binding was blocked by incubating with a blocking buffer (Sigma) for 30 min. After washing with PBST (0.1% Tween 20 in PBS), human serum in serial two-fold dilutions from 1:20 to 1:2560 (50 μL/well) was added to the plates and incubated at RT for 1h. After washing with PBST, the plates were incubated with 1:200 diluted solution of AP-linked goat anti-human IgG and IgM antibody (50 μL/well) at RT for 45 min. After washing, a p-nitrophenylphosphate solution (cat# P7998, Sigma) was added to the plates for 20 min and stopped by alkaline phosphatase stop solution (cat# A5852, Sigma). The signal was read at 405 nm wavelength by a spectrophotometer (Multiskan Spectrum, Thermo Fisher Scientific).

Immunohistochemistry

After deparaffinization and rehydration, tissue sections were treated with *AR-10* solution (HK057-5K, BioGenex) by autoclave at 121°C for 5 min. The endogenous peroxidase activity was quenched with 3% H_2O_2/PBS for 15 minutes followed by washing the slide in PBST for 5 minutes. The sections were incubated with anti-Globo H antibody VK9 or isotype control (401302, BioLegend) in a humidified chamber overnight at 4°C. After washing, sections were incubated with the Super Enhancer^{TM} reagent for 30 min and then the Poly-HRP reagent for 30 min. DAB substrate solution was used to detect Globo H staining signal (2-5 minutes). Finally, the sections were counterstained with Harris' Hematoxylin (cat#1.09253.2500, Merck).

Immunophenotyping

Blood collection using sodium-heparin blood tube from 71 patients in UCSF and 28 patients in CGMH-LK at baseline (pre-vaccine), day 4 (3 days post 1st administration of CY and prior to first vaccination), week 5 (4 weeks after first CY and before second CY), week 13 (12 weeks after first CY, intermediate time point), and week 41 (40 weeks after first CY) were for immune cell staining. Peripheral blood mononuclear cells (PBMC) were isolated using Ficoll-Paque (GE Healthcare) density centrifugation. Aliquots of blood cells were stained with antibodies to CD4 (BD Pharmingen), CD45RA (BD Pharmingen), and CD25 (BD Pharmingen) for 30 min at 4 degree C. Following the washing, fixation and permeabilization process, the cells were stained with antibody to FOXP3 (eBioscience) for 30 min to examine total Treg ($CD4^+Foxp3^+$, $CD4^+CD25^+$ or $CD4^+CD25^+Foxp3^+$), resting Treg ($CD45RA^+Foxp3^{low}$), activated Treg ($CD45RA^-Foxp3^{high)}$, and non-suppressive Treg ($CD45RA^-Foxp3^{low}$) [Miyara *et. al.*, 2009]. The corresponding negative control staining were mouse IgG1k-PerCP, mouse IgG2b-FITC, mouse IgG1k-PE, and rat IgG2a, k-APC, respectively. For NK ($CD3^-CD56^+$) /NKT ($CD3^+CD56^+$) cell staining, aliquots of PBMC were stained

with antibodies to CD3 (BD Biosciences), CD56 (BD Biosciences), and CD107a (BD Biosciences) or CD69 (BD Biosciences) for 30 min on ice. The expression of CD107a or CD69 on NK or NKT cells were examined. For Th1/Th2/Th17/CD8 Tc cell activation and staining, aliquots of PBMC were first incubated in 10%FBS/RPMI medium at 37 degree C for overnight. Following the protocol of Human Th1/Th2/Th17 Phenotyping Kit (BD Pharmingen) for stimulation and staining, the PBMC were stimulated with 50ng/ml phorbol-12-myristate-13-acetate plus 1 μg/ml ionomycin, or left unstimulated, for 1 hour and then Golgi Stop for 4 hours. The cells were further stained with antibodies to CD4, IL-17a, IL-4 and IFN-γ or CD3, CD8 and IFN-γ for 30 min at room temperature to examine Th1 ($CD4^+IFN\text{-}\gamma^+$), Th2 ($CD4^+IL4^+$), Th17 ($CD4^+IL17^+$) and circulating cytotoxic $CD8^+$ T cells ($CD3^+CD8^+IFN\text{-}\gamma^+$). The stained cells were analyzed by flow cytometry on a FACSCalibur, and changes in the different Th subsets, Treg, Tc, NK, and NKT cell populations were evaluated over time.

Supplemental Figures

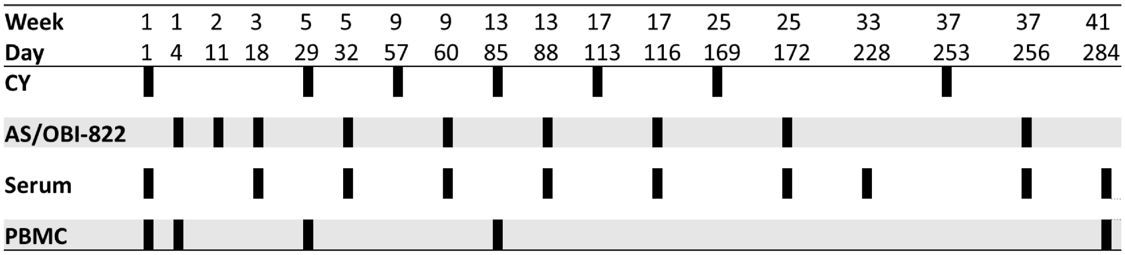

Supplemental figure S1 Treatment schema of cyclophosphamide and AS/OBI-821 and sampling schedule. Prior to drug administration, serum and peripheral blood mononuclear cell (PBMC) were collected for ELISA and immunophenotyping, respectively. CY: cyclophosphamide.

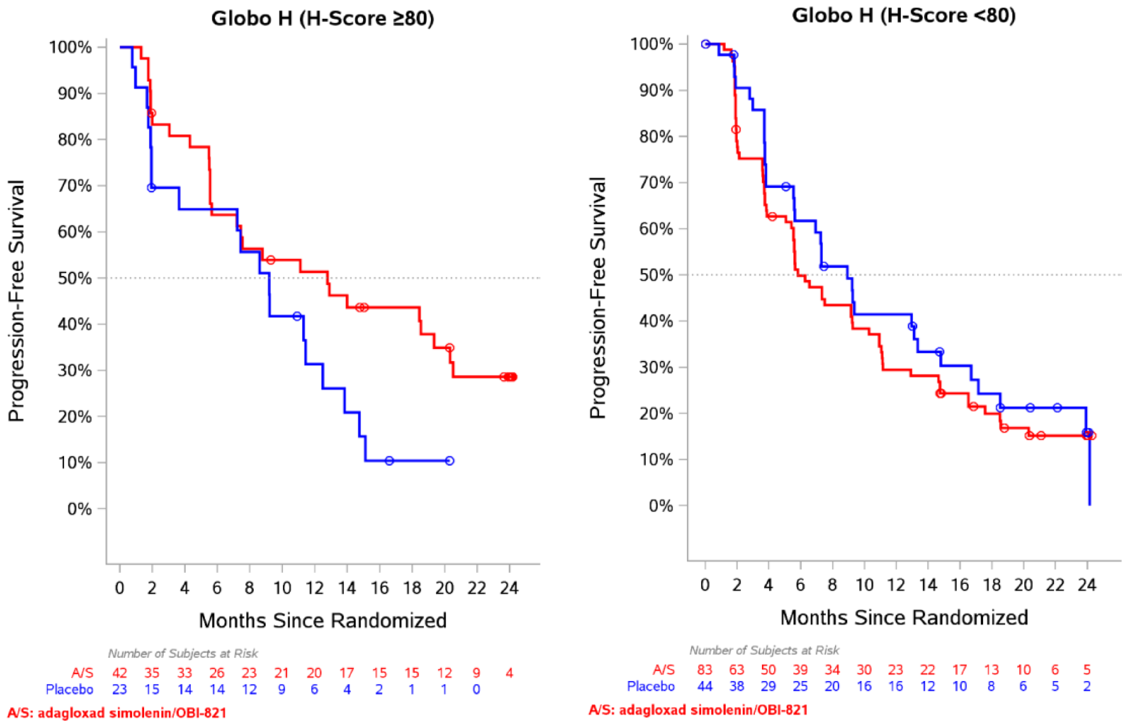

Supplemental figure S2 Progression-free survival of subjects by Globo H expression

Tumor tissue samples (primary site, n=192) were used to examine the expression of Globo H. Survival curves of the vaccinated (A/S, red line) and placebo (blue line) groups were stratified by Globo H expression at H-score of 80 (Left panel, H score ≥ 80; right panel, H score < 80).

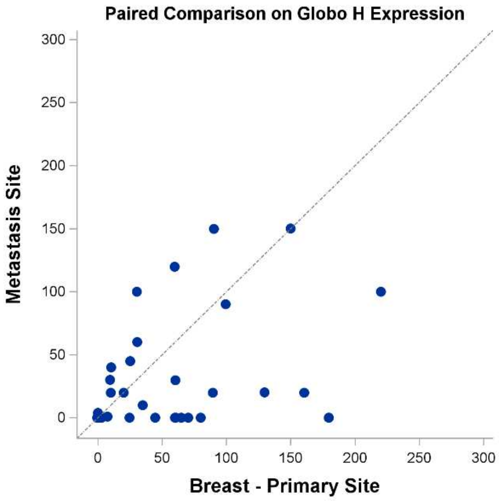

Supplemental figure S3 Globo H expression at metastatic site vs. primary site

Paired primary breast tumor tissue and metastatic tissue samples from the same subject were used to examine the expression of Globo H.

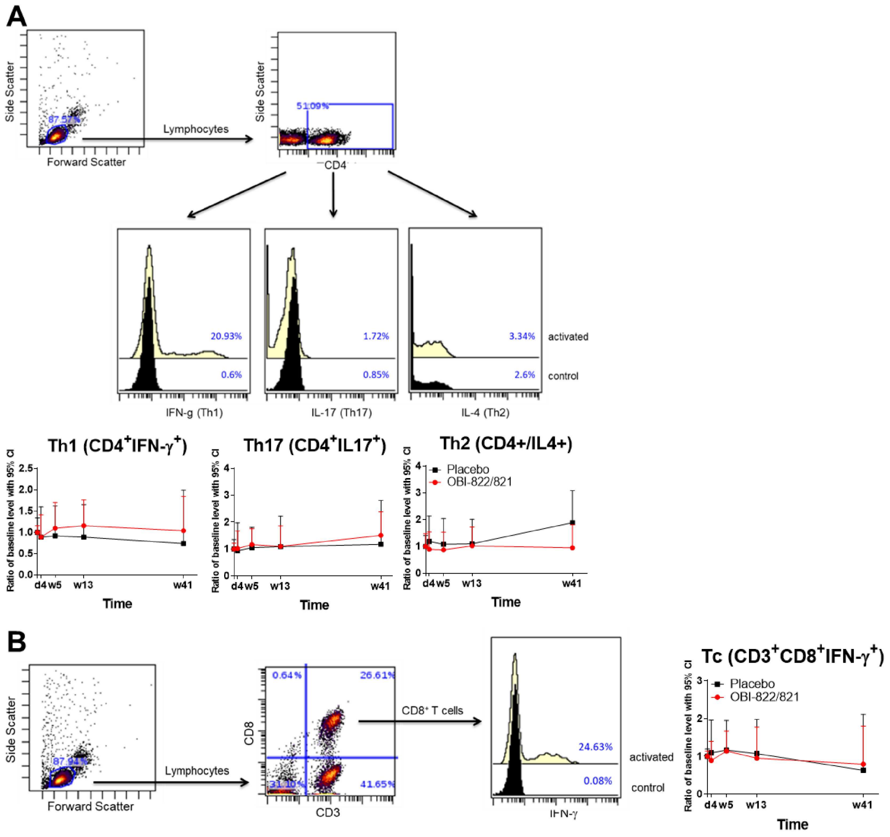
A
Side Scatter
Forward Scatter
Lymphocytes
CD4
51.09%
IFN-g (Th1)
IL-17 (Th17)
IL-4 (Th2)
20.93%
0.6%
1.72%
0.85%
3.34%
2.6%
activated
control
Th1 (CD4+IFN-γ+)
Th17 (CD4+IL17+)
Th2 (CD4+/IL4+)
Placebo
OBI-822/821
Ratio of baseline level with 95% CI
Time
d4 w5 w13 w41
B
CD8
CD3
0.64%
26.61%
41.65%
CD8+ T cells
24.63%
0.08%
IFN-γ
Tc (CD3+CD8+IFN-γ+)

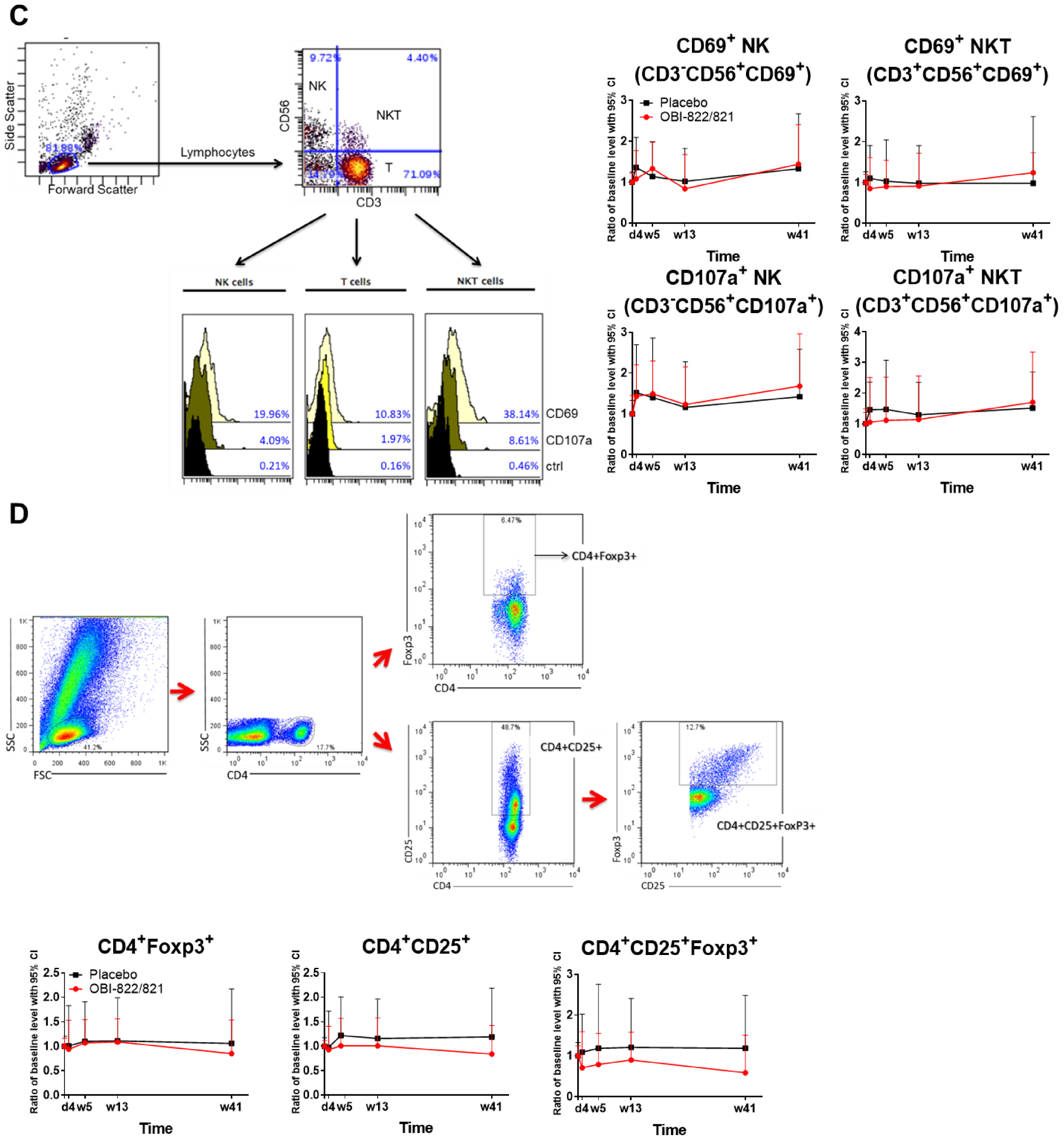
C
Side Scatter
Forward Scatter
Lymphocytes
CD56
CD3
NK
NKT
T
9.72%
4.40%
71.09%
NK cells
T cells
NKT cells
19.96%
4.09%
0.21%
10.83%
1.97%
0.16%
38.14%
8.61%
0.46%
CD69
CD107a
ctrl
CD69+ NK (CD3-CD56+CD69+)
CD69+ NKT (CD3+CD56+CD69+)
CD107a+ NK (CD3-CD56+CD107a+)
CD107a+ NKT (CD3+CD56+CD107a+)
Ratio of baseline level with 95% CI
Placebo
OBI-822/821
Time
d4 w5 w13 w41
D
SSC
FSC
CD4
Foxp3
CD25
6.47%
48.7%
12.7%
17.7%
41.2%
CD4+Foxp3+
CD4+CD25+
CD4+CD25+FoxP3+
CD4+Foxp3+
CD4+CD25+
CD4+CD25+Foxp3+

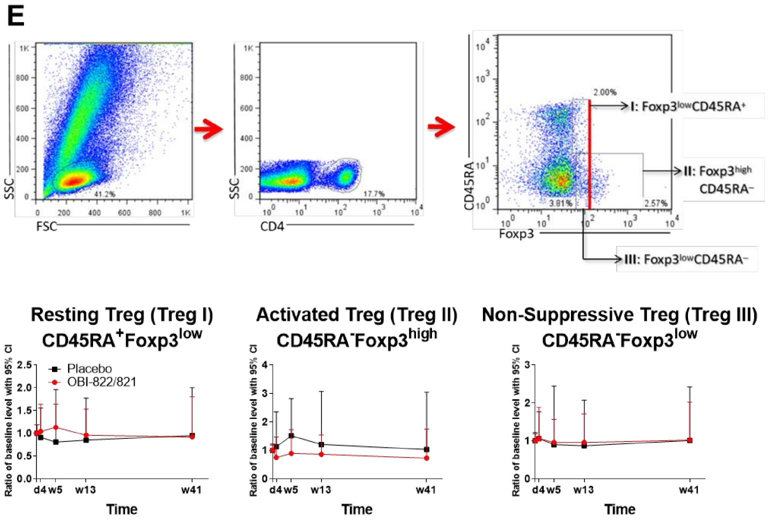

Supplemental figure S4. Circulating immune cell subpopulation after cyclophosphamide administration and immunization with AS/OBI-821 PBMC from patients were isolated before (baseline), after 1st CY administration (D4) and afterW4, W13, W41 immunization for immune marker analysis by FACS. Definition of Th subsets (A), Tc (B), NK and NKT (C), total Treg population (D), and Treg subsets (E), were shown. Ratio of baseline level with 95% confidence interval over time in placebo and AS/OBI-821 groups were presented.

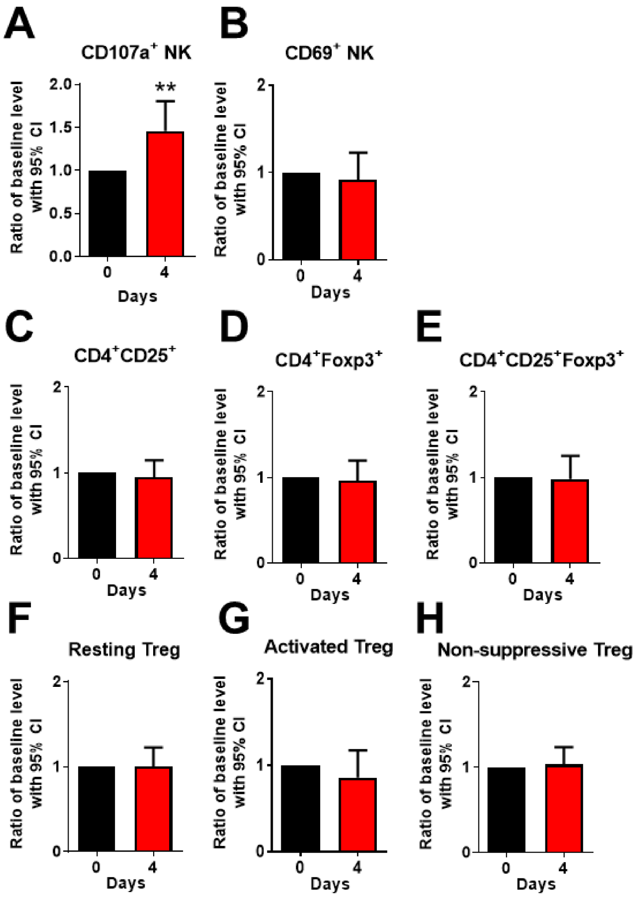

Supplemental figure S5. **Impact of Cyclophosphamide on NK and Treg**

PBMC from patients were isolated before (baseline) and after 1st CY administration (D4) for immune marker analysis by FACS for (A) CD107a$^+$ NK (CD3$^-$CD56$^+$CD107a$^+$), (B) CD69$^+$ NK (CD3$^-$CD56$^+$CD69$^+$), (C) CD4$^+$CD25$^+$ Treg, (D) CD4$^+$Foxp3$^+$ Treg, (E) CD4$^+$CD25$^+$Foxp3$^+$ Treg, (F) resting Treg (Treg I, CD45RA$^+$Foxp3^{low}), (G)activated Treg (Treg II, CD45RA$^-$Foxp3^{high}), and (H) Non-suppressive Treg (CD45RA$^-$Foxp3^{low}). The ratio of baseline level with 95% confidence interval on day 0 (black bar) and 4 (red bar) in AS/OBI-821 group were normalized to the ones in placebo group, respectively.

Reference

Miyara M, Yoshioka Y, Kitoh A, Shima T, Wing K, Niwa A, Parizot C, Taflin C, Heike T, Valeyre D, Mathian A, Nakahata T, Yamaguchi T, Nomura T, Ono M, Amoura Z, Gorochov G, Sakaguchi S. 2009 Functional delineation and differentiation dynamics of human CD4+ T cells expressing the Foxp3 transcription factor. Immunity. 19; 30(6):899-911.
